# The Mt Halimun-Salak Malaise Trap project - releasing the most species rich DNA Barcode library for Indonesia

**DOI:** 10.3897/BDJ.6.e29927

**Published:** 2018-12-19

**Authors:** Bruno Cancian de Araujo, Stefan Schmidt, Olga Schmidt, Thomas von Rintelen, Rosichon Ubaidillah, Michael Balke

**Affiliations:** 1 SNSB-Zoologische Staatssammlung München, Munich, Germany SNSB-Zoologische Staatssammlung München Munich Germany; 2 Museum für Naturkunde, Leibniz-Institut für Evolutions- und Biodiversitätsforschung, Berlin, Germany Museum für Naturkunde, Leibniz-Institut für Evolutions- und Biodiversitätsforschung Berlin Germany; 3 Museum Zoologicum Bogoriense, Research Center for Biology, Indonesian Institute of Sciences, Cibinong, Indonesia Museum Zoologicum Bogoriense, Research Center for Biology, Indonesian Institute of Sciences Cibinong Indonesia

**Keywords:** Biodiversity, BOLD, Chalcidoidea, Coleoptera, Diptera, Hymenoptera, IndoBioSys, Inventory, Java, Lepidoptera

## Abstract

The Indonesian archipelago features an extraordinarily rich biota. However, the actual taxonomic inventory of the archipelago remains highly incomplete and there is hardly any significant taxonomic activity that utilises recent technological advances. The IndoBioSys project was established as a biodiversity information system aiming at, amongst other goals, creating inventories of the Indonesian entomofauna using DNA barcoding. Here, we release the first large scale assessment of the megadiverse insect groups that occur in the Mount Halimun-Salak National Park, one of the largest tropical rain-forest ecosystem in West Java, with a focus on Hymenoptera, Coleoptera, Diptera and Lepidoptera collected with Malaise traps. From September 2015 until April 2016, 34 Malaise traps were placed in different localities in the south-eastern part of the Halimun-Salak National Park. A total of 4,531 specimens were processed for DNA barcoding and in total, 2,382 individuals produced barcode compliant records, representing 1,195 exclusive BINs or putative species in 98 insect families. A total of 1,149 BINs were new to BOLD. Of 1,195 BINs detected, 804 BINs were singletons and more than 90% of the BINs incorporated less than five specimens. The astonishing heterogeneity of BINs, as high as 1.1 exclusive BIN per specimen of Diptera successfully processed, shows that the cost/benefit relationship of the discovery of new species in those areas is very low. In four genera of Chalcidoidea, a superfamily of the Hymenoptera, the number of discovered species was higher than the number of species known from Indonesia, suggesting that our samples contain many species that are new to science. Those numbers shows how fast molecular pipelines contribute substantially to the objective inventorying of the fauna giving us a good picture of how potentially diverse tropical areas might be.

## Introduction

The Indonesian archipelago features an extraordinarily rich biota that is, amongst other factors, derived from its sheer size and geographic position, basically linking the Oriental and Australian regions. This transition was first described in detail by Wallace (1860), who laid the foundation for the discipline of biogeography in this region. Our understanding of the biogeography of the region has steadily advanced since then, increasingly embracing new technology and interdisciplinary research approaches (see Lohman et al. 2011). However, the actual taxonomic inventory of the archipelago remains highly incomplete (see Schmidt 2015) and there is hardly any significant taxonomic activity that utilises recent technological advances (but see Barlow and Woiwod 1990, Riedel et al. 2013, Riedel et al. 2014, Wibowo et al. 2017, Hubert et al. 2015, Dahruddin et al. 2016, Cancian de Araujo et al. 2017, Cancian de Araujo et al. 2018). Large-scale databasing, in particular of hyperdiverse invertebrates of the region, is also in its infancy. The GBIF to date only features 147,463 occurrence data published for Indonesia, for 13,210 species - surprisingly few compared, for example, to Germany with 37,917,568 occurrences and 16,742 species (GBIF, accessed on 1 July 2018). At the same time, vast areas of supposedly high biodiversity disappear every year (Brooks et al. 2002, Curran et al. 2004, Gaveau et al. 2013, Wilcove et al. 2013, Abood et al. 2014, Margono et al. 2014) and with them, possibly thousands of species never formally known to mankind, which means also a significant loss of ecosystem service and knowledge of potentially useful compounds (see Hooper et al. 2005, Loreau 2009, Norris 2011).

The Indonesian and German ministries of Research and Education have therefore provided funding to establish a biodiversity information system (IndoBioSys), that integrates occurrence databasing, species discovery and species characterisation, using morphology and DNA sequence data, specimen vouchering, as well as integrated tools for the discovery of substances of potential use for society. IndoBioSys is, therefore, a case study and foundation for the large-scale exploration of Indonesian species diversity. Moreover, IndoBioSys could be a foundation for the empirical and objective,scientific assessment of species distribution patterns across the archipelago, for example, needed for conservation priority setting.

One work package of the IndoBioSys project was an assessment of the species diversity of the hyperdiverse insect fauna of the Mount Halimun-Salak National Park in West Java, with a focus on sampling with Malaise traps. The National Park has been recognised as one of the largest tropical rain-forest ecosystems left in Java, being designated as a National Park in 2003 with a present area of about 113,357 hectares. Malaise trapping (Malaise 1937, Townes 1972) is a method that allows standardised sampling of flying insects, with a number of highly diverse groups of minute species, e.g. in the Diptera and Hymenoptera.

Subsets of the samples obtained were submitted to a well-established pipeline employing DNA barcoding (Hebert et al. 2003, Ivanova et al. 2006) in order to estimate species diversity (see Ratnasingham and Hebert 2007, Ratnasingham and Hebert 2013) and to obtain data for future beta diversity studies with data from other localities.

Here, we release these data with an analysis of their taxonomic content, an approximation of the species diversity encountered and an evaluation of the novelty of the data with respect to publicly available data from the Barcode of Life Data Systems (BOLD).

## Materials and Methods

A summary of fieldwork and laboratory procedures employed in the IndoBioSys project were presented by Schmidt et al. 2017. Methodological steps specific for the work package presented here are described below.

### Fieldwork and samples processing

**Figure 1.**
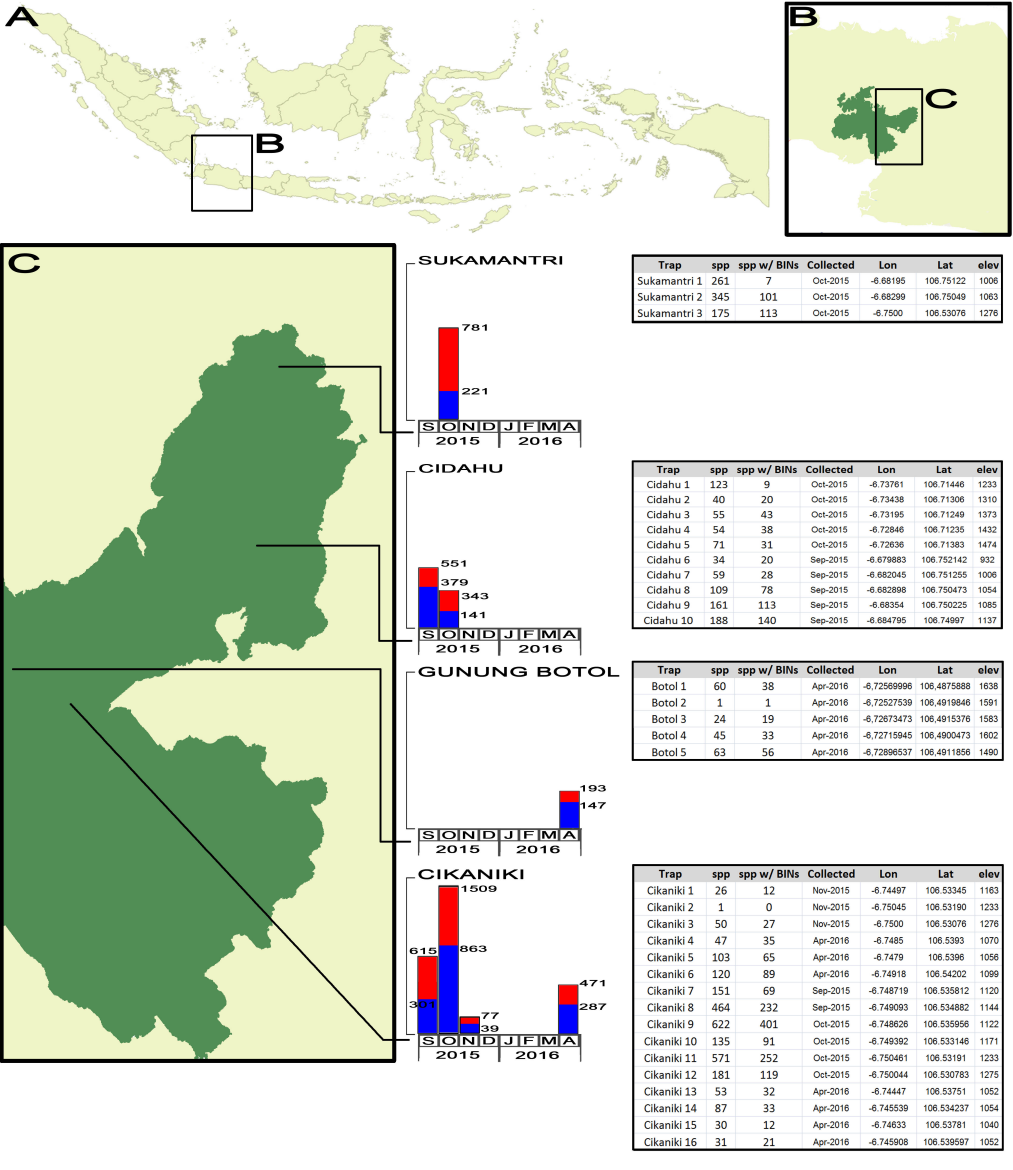
Spatial and temporal specimens and molecular access success distribution on Mt Halimun-Salak National Park.

From September 2015 until April 2016, 34 Malaise traps (Townes style, Townes 1972) were placed in four different localities in the southeast of the Halimun-Salak National Park (Fig. 1). The elevation ranged from 932 to 1,638 m with an average of 1,218 m.

The traps were run for about 120 days in total and the collecting bottles changed monthly. Collecting liquid was 300 ml of 96% Ethanol in each bottle.

The samples were taken to the IndoBioSys Indonesian laboratory at the Museum Zoologicum Bogoriense (MZB) in Cibinong, West Java. Using a 3 mm mesh sieve, they were broken down into two fractions, according to the size of the animals with the smaller samples passing the sieve into a collecting tray.

This fractioning is important for optimising the sorting process as well as for separating the specimens that will be sent entirely for molecular laboratory processing ("voucher recovery pipeline") from the ones that are large enough for a procedure where only one or more legs are removed from the voucher for laboratory use ("leg picking pipeline"). Most of the fractions were sent to the IndoBioSys laboratory at SNSB-ZSM in Munich, where they were sorted to order and family level.

Given the enormous number of specimens (we estimated over 300,000 specimens of invertebrates collected during the project), the orders Coleoptera and Hymenoptera were chosen as the main target groups for the present analysis. Selected groups of Diptera, in particular Syrphidae and Phoridae, will be dealt with in a separate data release. Here, we present the results of a few specimens randomly picked from the samples. For Coleoptera and Hymenoptera, specimens were taken quantitatively from the samples except in case of a long series of morphologically similar individuals, in which case we took only representatives. In these cases, a smaller amount of specimens that represents the morphological diversity of the series was chosen in order to prevent cryptic species bias. The number of specimens taken was determined on a case by case basis.

Lepidoptera, another target group of the IndoBioSys project, were collected using a different method, as described earlier (Schmidt et al. 2017). Some Geometridae that were collected using Malaise traps and that were suitable for morphological analysis were processed and included in the present study. A specific release of the geometrid barcode data is currently being prepared (OS in prep.).

**Figure 2.**
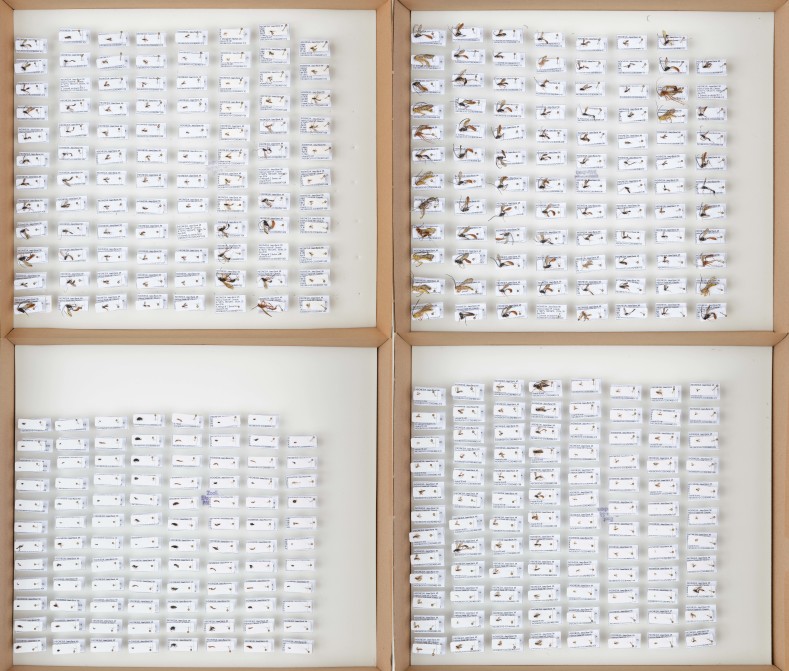
Voucher specimens of the IndobioSys project, mounted and labelled for repatriation to the MZB.

**Figure 3.**
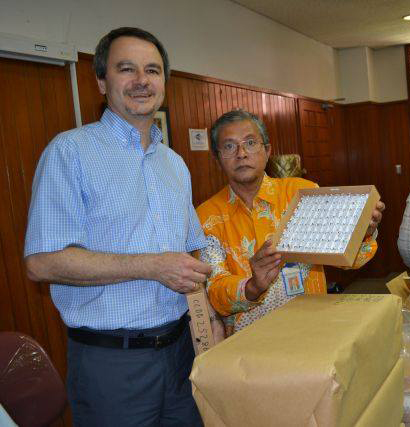
Dr Michael Balke (ZSM, Munich) and Dr Rosichon Ubaidillah (MZB) at the Museum Zoologicum Bogoriense in Cibinong, West Java, during the repatriation of over 2,000 voucher specimens and over 20 ethanol samples.

All specimens that were not further processed were repatriated to the MZB as ethanol samples. All processed specimens were returned to MZB as dry mounted and labelled voucher specimens (Fig. 2 and Fig. 3).

All specimen data are accessible in BOLD as a single citable dataset (dx.doi.org/10.5883/DS-IDBMTP). The data include collecting locality, geographic coordinates, elevation, collector, one or more digital images, identifier and voucher depository. Sequences data can be obtained through BOLD and include a detailed LIMS report, primer information and access to trace files. The sequences are also available on GenBank (accession numbers MH926363-MH929079).

### Data analysis

Locality information and molecular data from the Malaise trapping programme were downloaded from the BOLD IndoBioSys campaign projects. The records downloaded were individualised by trap and by insect order in separate excel worksheets for analysis of spatial and diversity distribution. Here, we only focus on the orders Hymenoptera, Coleoptera, Diptera and Lepidoptera.

## Results

A total of 4,531 specimens were prepared for DNA barcoding. Of these, we obtained cox1-5P sequences from 2,732 individuals. Sequences from 2,598 of these individuals were longer than 300 base pairs. In total, 2,380 individuals produced barcode compliant records (Table 1). The success rate was therefore comparably low, with only 60.5% on average, varying between the samples from 2.7% to 100% (Fig. 1).

**Figure 4.**
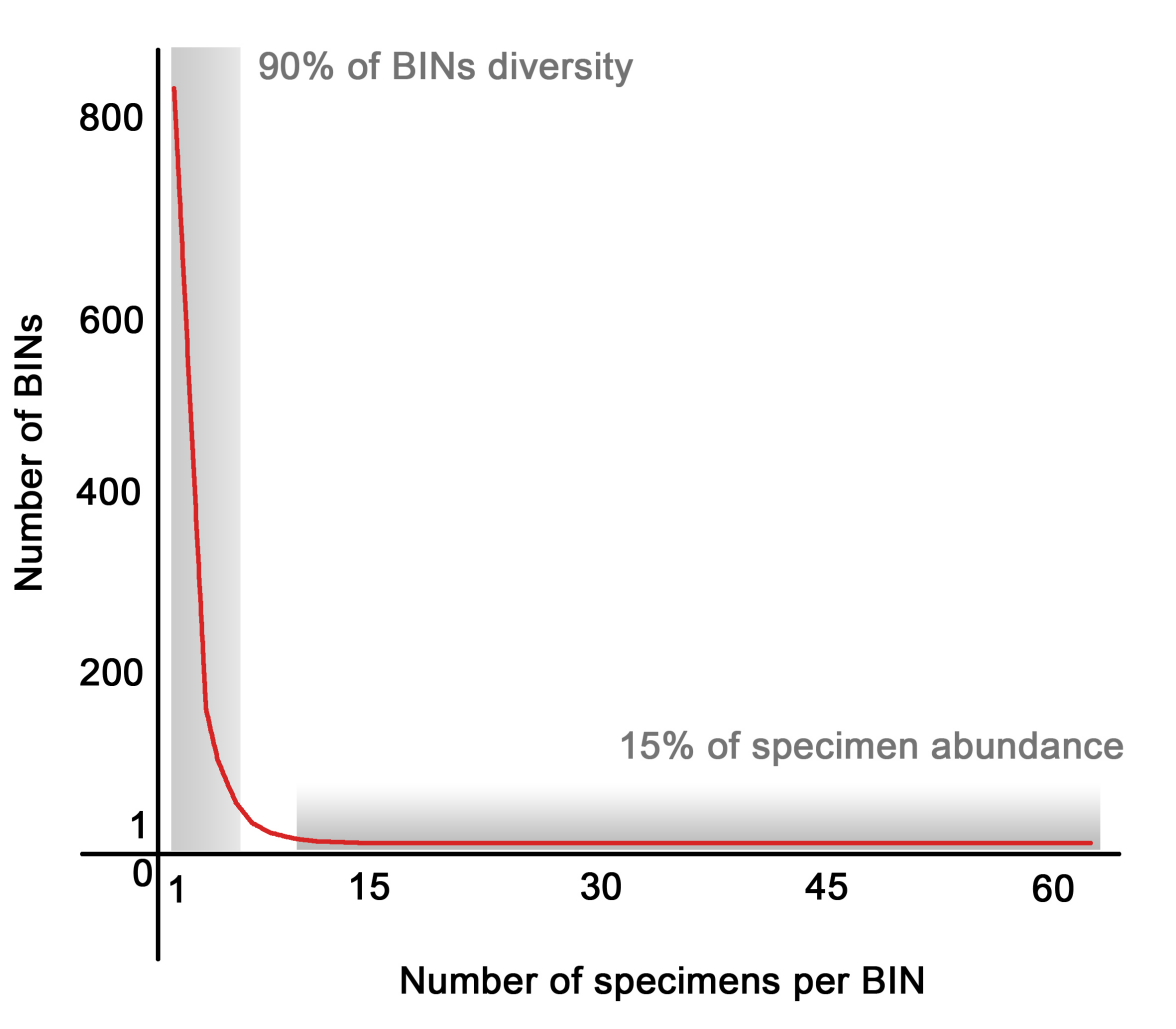
BINs Diversity and relative abundance

These 2,380 individuals represent 1,197 exclusive BINs or putative species. They could be assigned to 98 different insect families (Table 2). Gunung Botol had the largest success rate (80.9%) and Sukamantri the lowest (32.2%) in terms of processed specimens producing barcode compliant sequences. From those 1,197 exclusive BINs, only 46 BINs (3.8%) are not new to BOLD. Only 15 BINs were recovered with more than 10 specimens of each BIN. A total of 804 BINs were singletons and more than 90% of the BINs were recorded with less than 5 specimens (Fig. 4).

The highest diversity of BINs was found in Hymenoptera (712 BINs), followed by Coleoptera (398), Diptera (53) and Lepidoptera (34). The diversity per order was always high, with two or less individuals per BIN on average. The diversity per family was also impressive with 50% of the families being composed by BINs represented by singletons or doubletons.

## Discussion

Given the discrepancy in the sampling effort, it was not possible to compare taxonomic disparities amongst the four sampling areas. The sampling was focused on Cikaniki due to the better conservation of the forest in this area and the presence of the research station that provided better infrastructure to the scientific staff.

**Figure 5.**
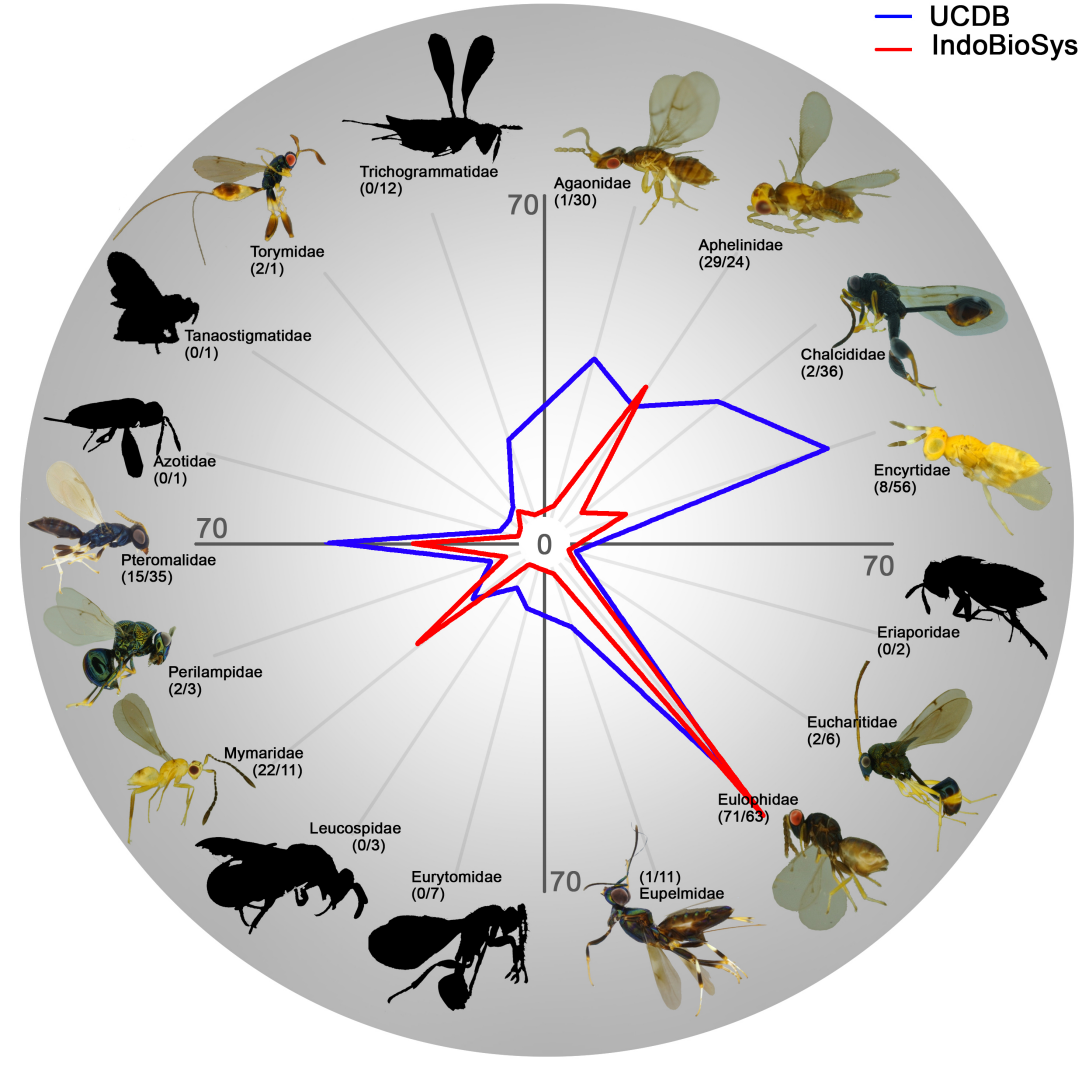
IndoBioSys Chalcidoidea species diversity per family (red line) compared to the Universal Chalcidoidea Database (UCDB) species diversity (blue line). The number of species is presented between parenthesis close to the family name (IndoBioSys / UCDB).

Even collecting at four different locations in one nature reserve, the IndoBioSys Malaise trap project alone has added 1,149 new BINs to BOLD. It shows how fast molecular pipelines contribute substantially to objectively inventorying the fauna of megadiverse areas. It also allows us to estimate the enormous diversity of tropical areas like the Halimun-Salak National Park. The astonishing heterogeneity of BINs (See Fig. 5 and Table 2), as high as 1.1 specimen successfully processed per exclusive BIN of Diptera, shows the magnitude of the diversity that is waiting to be discovered in the tropics. Only 15% of the specimens that produced DNA barcode compliant records belong to putative species that have more than five specimens processed, being 81.7% of all BINs represented by singletons or doubletons. It makes the cost/benefit relationship of the discovery of new species in those areas very low, even with low success rates of the molecular processing that this project has been facing. Such large error rates have not been encountered in similar projects of the ZSM and we suspect that the poor quality of the ethanol used for the collecting bottles might have been the crucial issue.

The supraspecific taxonomic diversity was relatively high considering the number of specimens analysed. As a comparison, Hendrich and collaborators in their release of a comprehensive DNA barcode database for Central European beetles (Hendrich et al. 2014) have sequenced 15,948 specimens to obtain 97 families meaning that, on average, a family in the database is represented by 164.4 processed specimens. In the present paper, we recorded 39 families of Coleoptera after processing only 788 specimens, corresponding to one family per 20.7 specimens on average. Therefore and even considering that this discovery process is not linear, it is quite clear that we are far behind the accumulation curve plateau for families and that there are many more to be discovered at Halimun-Salak National Park, especially at the species level.

The diversity of Chalcidoidea, a superfamily of Hymenoptera, gives us a clear picture of the diversity uncovered at Halimun-Salak National Park. The Universal Chalcidoidea Database (Noyes 2018) has returned records for 17 genera and 302 species from Java. Here, we detected 11 genera and 155 species for this superfamily. For four families (Aphelinidae, Eulophidae, Mymaridae and Torymidae), the diversity detected was higher than the diversity described (Fig. 5), showing that those samples are composed of many species new to science.

## Figures and Tables

**Table 1.** Specimens and BINs distribution per order.

**Order**	**Total indiv.**	%	**Indiv. with** **sequence**	%	**BINs**	%	**Proccessed indiv. per BIN**	**Indiv. w** **sequence per BIN**
Diptera	108	2.4	67	62.0	53	4.4	2.2	1.1
Lepidoptera	169	3.7	132	78.1	34	2.8	5.0	3.9
Coleoptera	1,174	25.9	835	71.1	398	33.2	2.9	2.1
Hymenoptera	3,080	68.0	1,702	55.3	712	59.5	4.3	2.4

**Table 2.** Specimens and BINs distribution per Family

** COLEOPTERA **				** HYMENOPTERA **			
**Family**	**spp w/ BINs**	**Unique BINs**	**spp/BIN**	**Family**	**spp w/ BINs**	**Unique BINs**	**spp/BIN**
Brentidae	1	1	1.0	Agaonidae	5	1	5.0
Buprestidae	1	1	1.0	Aphelinidae	36	29	1.2
Erotylidae	1	1	1.0	Braconidae	223	139	1.6
Ptiliidae	1	1	1.0	Chalcididae	2	2	1.0
Tetratomidae	1	1	1.0	Crabronidae	20	1	20.0
Anthicidae	2	1	2.0	Encyrtidae	12	8	1.5
Carabidae	2	1	2.0	Eucharitidae	8	2	4.0
Cerambycidae	2	2	1.0	Eulophidae	133	71	1.9
Melandryidae	2	2	1.0	Eupelmidae	2	1	2.0
Limnichidae	3	1	3.0	Ichneumonidae	881	370	2.4
Attelabidae	3	3	1.0	Mymaridae	32	22	1.5
Leiodidae	3	3	1.0	Perilampidae	2	2	1.0
Mycetophagidae	3	3	1.0	Pteromalidae	50	15	3.3
Nitidulidae	3	3	1.0	Sphecidae	3	1	3.0
Ptinidae	3	3	1.0	Tenthredinidae	3	1	3.0
Eucnemidae	4	2	2.0	Torymidae	6	2	3.0
Tenebrionidae	4	3	1.3	Unknown	63	44	1.5
Throscidae	7	4	1.8	Vespidae	7	1	7.0
Melyridae	7	6	1.2	**TOTAL**	**1,488**	**712**	**2.2**
Anthribidae	9	7	1.3				
Scarabaeidae	11	5	2.2	** LEPIDOPTERA **			
Scirtidae	11	5	2.2	**Family**	**spp w/ BINs**	**Unique BINs**	**spp/BIN**
Cleridae	14	4	3.5	Geometridae	25	17	1.5
Latridiidae	15	3	5.0	Noctuidae	1	1	1
Curculionidae	15	11	1.4	Erebidae	1	1	1
Lampyridae	16	3	5.3	Uraniidae	1	1	1
Lycidae	16	11	1.5	Unknown	23	14	1.6
Aderidae	19	6	3.2	**TOTAL**	**51**	**34**	**1.2**
Phalacridae	20	8	2.5				
Elateridae	20	11	1.8	** DIPTERA **			
Mordellidae	22	15	1.5	**Family**	**spp w/ BINs**	**Unique BINs**	**spp/BIN**
Corylophidae	24	11	2.2	Phoridae	5	5	1.0
Hydrophilidae	28	3	9.3	Muscidae	3	2	1.5
Scraptiidae	37	7	5.3	Cecidomyiidae	1	1	1.0
Cantharidae	42	15	2.8	Tipulidae	1	1	1.0
Chrysomelidae	69	36	1.9	Syrphidae	1	1	1.0
Coccinellidae	71	35	2.0	Asilidae	2	2	1.0
Staphylinidae	114	35	3.3	Tachinidae	1	1	1.0
Unknown	154	126	1.2	Unknown	47	40	1.2
**TOTAL**	**780**	**398**	**2.2**	**TOTAL**	**61**	**53**	**1.2**

## References

[B4502596] Abood Sinan A., Huay Lee Janice Ser, Burivalova Zuzana, Garcia-Ulloa John, Koh Lian Pin (2014). Relative contributions of the logging, fiber, oil palm, and mining industries to forest loss in Indonesia. Conservation Letters.

[B4423329] Barlow H. S., Woiwod I. P., Knight W. J., Holloway J. D. (1990). Seasonality and diversity of Macrolepidoptera in two lowland sites in Dumoga-Bone National Park, Sulawesi Utara. Insects and the Rain Forest of South East Asia (Wallacea).

[B4502607] Brooks Thomas M., Mittermeier Russell A., Mittermeier Cristina G., da Fonseca Gustavo A. B., Rylands Anthony B., Konstant William R., Flick Penny, Pilgrim John, Oldfield Sara, Magin Georgina, Hilton-Taylor Craig (2002). Habitat Loss and Extinction in the Hotspots of Biodiversity. Conservation Biology.

[B4423186] Cancian de Araujo Bruno, Schmidt Stefan, von Rintelen Thomas, Sutrisno Hari, von Rintelen Kristina, Ubaidillah Rosichon, Häuser Chrisoph, Djunijanti Peggie, Narakusumo R. P., Balke Michael (2017). IndoBioSys – DNA barcoding as a tool for the rapid assessment of hyperdiverse insect taxa in Indonesia: a status report. Treubia.

[B4423202] Cancian de Araujo Bruno, Schmidt Stefan, Schmidt Olga, Rintelen Thomas von, Kilmaskossu Agustinus, Panjaitan Rawati, Balke Michael (2018). From field courses to DNA barcoding data release for West Papua - making specimens and identifications from university courses more sustainable. Biodiversity Data Journal.

[B4502568] Curran L. M., Trigg S. N., McDonald A. K., Astiani D., Hardiono Y. M., Siregar P., Caniago I., Kasischke E. (2004). Lowland forest loss in protected areas of Indonesian Borneo. Science.

[B4423172] Dahruddin Hadi, Hutama Aditya, Busson Frédéric, Sauri Sopian, Hanner Robert, Keith Philippe, Hadiaty Renny, Hubert Nicolas (2016). Revisiting the ichthyodiversity of Java and Bali through DNA barcodes: taxonomic coverage, identification accuracy, cryptic diversity and identification of exotic species. Molecular Ecology Resources.

[B4504608] Gaveau David L. A., Kshatriya Mrigesh, Sheil Douglas, Sloan Sean, Molidena Elis, Wijaya Arief, Wich Serge, Ancrenaz Marc, Hansen Matthew, Broich Mark, Guariguata Manuel R., Pacheco Pablo, Potapov Peter, Turubanova Svetlana, Meijaard Erik (2013). Reconciling forest conservation and logging in Indonesian Borneo. PLoS ONE.

[B4423293] Hebert P. D. N., Cywinska A., Ball S. L., deWaard J. R. (2003). Biological identifications through DNA barcodes. Proceedings of the Royal Society B: Biological Sciences.

[B4512645] Hendrich Lars, Morinière J, Haszprunar Gerhard, Hebert P. D. N., Hausmann Axel, Köhler Frank, Balke Michael (2014). A comprehensive DNA barcode database for Central European beetles with a focus on Germany: adding more than 3500 identified species to BOLD. Molecular Ecology Resources.

[B4502634] Hooper D. U., Chapin F. S., Ewel J. J., Hector A., Inchausti P., Lavorel S., Lawton J. H., Lodge D. M., Loreau M., Naeem S., Schmid B., Setälä H., Symstad A. J., Vandermeer J., Wardle D. A. (2005). Effects of biodiversity on ecosystem functioning: a consensus of current knowledge. Ecological Monographs.

[B4423128] Hubert Nicolas, Kadarusman, Wibowo Arif, Busson Frédéric, Caruso Domenico, Sulandari Sri, Nafiqoh Nuna, Pouyaud Laurent, Rüber Lukas, Avarre Jean-Christophe, Herder Fabian, Hanner Robert, Keith Philippe, Hadiaty Renny K. (2015). DNA Barcoding Indonesian freshwater fishes: challenges and prospects. DNA Barcodes.

[B4512832] Ivanova N. V., deWaard J. R., Hebert P. D. (2006). An inexpensive, automation-friendly protocol for recovering high-quality DNA. Molecular Ecology Notes.

[B4423304] Lohman D. J., de Bruyn M., Page Timothy, von Rintelen K., Hall Robert, Ng Peter K. L., Shih Hsi-Te, Carvalho G. R., von Rintelen Thomas (2011). Biogeography of the Indo-Australian Archipelago. Annual Review of Ecology, Evolution, and Systematics.

[B4502655] Loreau M. (2009). Linking biodiversity and ecosystems: towards a unifying ecological theory. Philosophical Transactions of the Royal Society B: Biological Sciences.

[B4506657] Malaise R. (1937). A new insect trap. Entomologisk Tidskrift.

[B4423252] Margono Belinda Arunarwati, Potapov Peter V., Turubanova Svetlana, Stolle Fred, Hansen Matthew C. (2014). Primary forest cover loss in Indonesia over 2000–2012. Nature Climate Change.

[B4502624] Norris K. (2011). Biodiversity in the context of ecosystem services: the applied need for systems approaches. Philosophical Transactions of the Royal Society B: Biological Sciences.

[B4504654] Noyes J. S. Universal Chalcidoidea Database. World Wide Web electronic publication. http://www.nhm.ac.uk/chalcidoids.

[B4512852] Ratnasingham S., Hebert P. D (2007). BOLD: The Barcode of Life Data System. Molecular Ecology Resources.

[B4512862] Ratnasingham S., Hebert P. D.N. (2013). A DNA-based registry for all animal species: The Barcode Index Number (BIN) system. PLoS ONE.

[B4687733] Riedel Alexander, Sagata Katayo, Suhardjono Yayuk R, Tänzler Rene, Balke Michael (2013). Integrative taxonomy on the fast track - towards more sustainability in biodiversity research. Frontiers in Zoology.

[B4687744] Riedel Alexander, Tänzler Rene, Balke Michael, Rahmadi Cahyo, Suhardjono Yayuk R. (2014). Ninety-eight new species of *Trigonopterus* weevils from Sundaland and the Lesser Sunda Islands. ZooKeys.

[B4423319] Schmidt Olga (2015). List of primary types of the larentiine moth species (Lepidoptera: Geometridae) described from Indonesia - a starting point for biodiversity assessment of the subfamily in the region. Biodiversity Data Journal.

[B4423215] Schmidt Olga, Hausmann Axel, Cancian de Araujo Bruno, Sutrisno Hari, Djunijanti Peggie, Schmidt Stefan (2017). A streamlined collecting and preparation protocol for DNA barcoding of Lepidoptera as part of large-scale rapid biodiversity assessment projects, exemplified by the Indonesian Biodiversity Discovery and Information System (IndoBioSys). Biodiversity Data Journal.

[B4506667] Townes H. (1972). A light-weight Malaise trap. Entomological News.

[B4423273] Wallace Alfred R. (1860). On the zoological geography of the Malay Archipelago.. Journal of the Proceedings of the Linnean Society of London. Zoology.

[B4423283] Wibowo Arif, Wahlberg Niklas, Vasemaegi Anti (2017). DNA barcoding of fish larvae reveals uncharacterised biodiversity in tropical peat swamps of New Guinea, Indonesia. Marine and Freshwater Research.

[B4502585] Wilcove David S., Giam Xingli, Edwards David P., Fisher Brendan, Koh Lian Pin (2013). Navjot's nightmare revisited: logging, agriculture, and biodiversity in Southeast Asia. Trends in Ecology & Evolution.

